# Poly[[diaqua­bis­{μ-4-[6-(4-carboxyphenyl)-4,4′-bipyridin-2-yl]benzoato-κ^2^ *O*:*N* ^4′^}zinc] dimethyl­formamide tetra­solvate]

**DOI:** 10.1107/S1600536812043838

**Published:** 2012-10-27

**Authors:** Xin Ge, Shuyan Song

**Affiliations:** a State Key Laboratory of Rare Earth Resource Utilization, Changchun Institute of Applied Chemistry, Chinese Academy of Sciences, Changchun 130022, People’s Republic of China

## Abstract

In the title compound, {[Zn(C_24_H_15_N_2_O_4_)_2_(H_2_O)_2_]·4C_3_H_7_NO}_*n*_, the Zn^II^ ion is located on an inversion center and is six-coordinated by two N atoms from two ligands, two carboxylate O atoms from two other symmetry-related ligands and two O atoms from two water mol­ecules, furnishing a slightly distorted octa­hedral geometry. The Zn^II^ atoms are connected by the bridging ligands into a layer parallel to (101). O—H⋯O hydrogen bonds link the layers and the dimethyl­formamide solvent mol­ecules. π–π inter­actions between the pyridine and benzene rings [centroid–centroid distances = 3.7428 (17) and 3.7619 (17) Å] and intra­layer O—H⋯O hydrogen bonds are also present.

## Related literature

For the design of transition metal complexes with supra­molecular structures, see: Li *et al.* (2011 ▶); Wang *et al.* (2010 ▶); Yang *et al.* (2007 ▶). For related structures, see: Song *et al.* (2012 ▶).
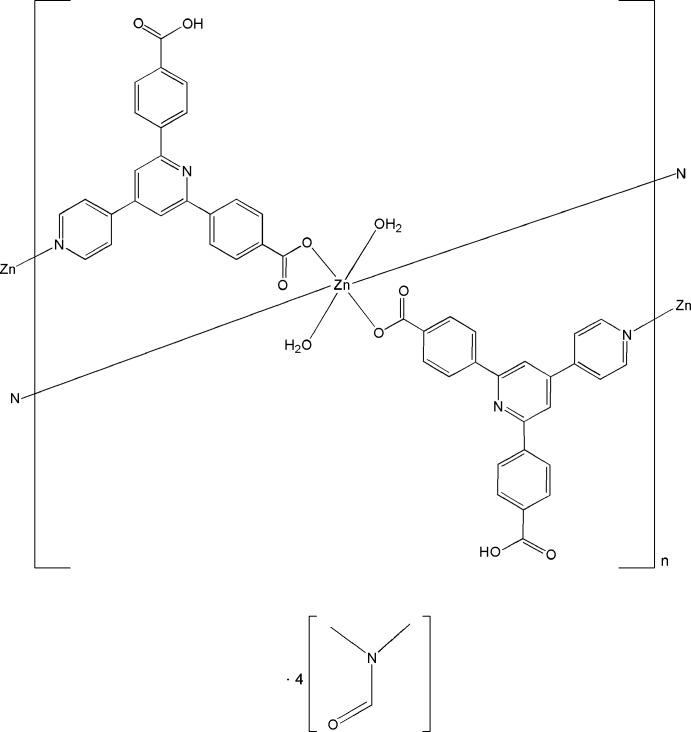


## Experimental

### 

#### Crystal data

[Zn(C_24_H_15_N_2_O_4_)_2_(H_2_O)_2_]·4C_3_H_7_NO
*M* *_r_* = 1184.57
Monoclinic, 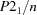

*a* = 7.4744 (5) Å
*b* = 17.7122 (13) Å
*c* = 21.4993 (15) Å
β = 95.075 (1)°
*V* = 2835.1 (3) Å^3^
*Z* = 2
Mo *K*α radiation
μ = 0.51 mm^−1^
*T* = 293 K
0.25 × 0.22 × 0.19 mm

#### Data collection

Bruker APEXII CCD diffractometer
Absorption correction: multi-scan (*SADABS*; Bruker, 2001 ▶) *T*_min_ = 0.839, *T*_max_ = 0.915
15534 measured reflections
5606 independent reflections
3804 reflections with *I* > 2σ(*I*)
*R*_int_ = 0.044

#### Refinement

*R*[*F* ^2^ > 2σ(*F* ^2^)] = 0.053
*wR*(*F* ^2^) = 0.158
*S* = 1.05
5606 reflections
385 parameters
3 restraints
H atoms treated by a mixture of independent and constrained refinement
Δρ_max_ = 0.40 e Å^−3^
Δρ_min_ = −0.35 e Å^−3^

### 

Data collection: *APEX2* (Bruker, 2007 ▶); cell refinement: *SAINT* (Bruker, 2007 ▶); data reduction: *SAINT*; program(s) used to solve structure: *SHELXTL* (Sheldrick, 2008 ▶); program(s) used to refine structure: *SHELXTL*; molecular graphics: *XP* in *SHELXTL* and *DIAMOND* (Brandenburg, 1999 ▶); software used to prepare material for publication: *SHELXTL* (Sheldrick, 2008 ▶).

## Supplementary Material

Crystal structure: contains datablock(s) global, I. DOI: 10.1107/S1600536812043838/hy2597sup1.cif

Structure factors: contains datablock(s) I. DOI: 10.1107/S1600536812043838/hy2597Isup2.hkl

Additional supplementary materials:  crystallographic information; 3D view; checkCIF report

## Figures and Tables

**Table 1:** Hydrogen-bond geometry (Å, °)

*D*—H⋯*A*	*D*—H	H⋯*A*	*D*⋯*A*	*D*—H⋯*A*
O1*W*—H1*A*⋯O6^i^	0.85 (1)	1.93 (2)	2.750 (4)	164 (4)
O1*W*—H1*B*⋯O2	0.85 (1)	1.98 (2)	2.718 (3)	145 (4)
O3—H3*A*⋯O2^ii^	0.86 (1)	1.77 (2)	2.592 (3)	161 (5)

Symmetry codes: (i) 
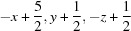
; (ii) 
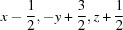
.

**Fig. 1.**
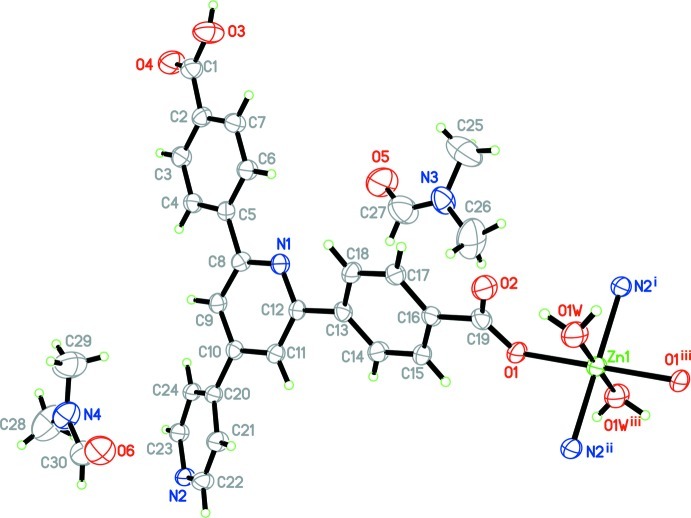
The asymmetric unit of the title compound. Displacement ellipsoids are drawn at the 50% probability level. [Symmetry codes: (i) 3/2-x, 1/2+y, 1/2-z; (ii) 1/2+x, 1/2-y, -1/2+z; (iii) 2-x, 1-y, -z.]

**Fig. 2.**
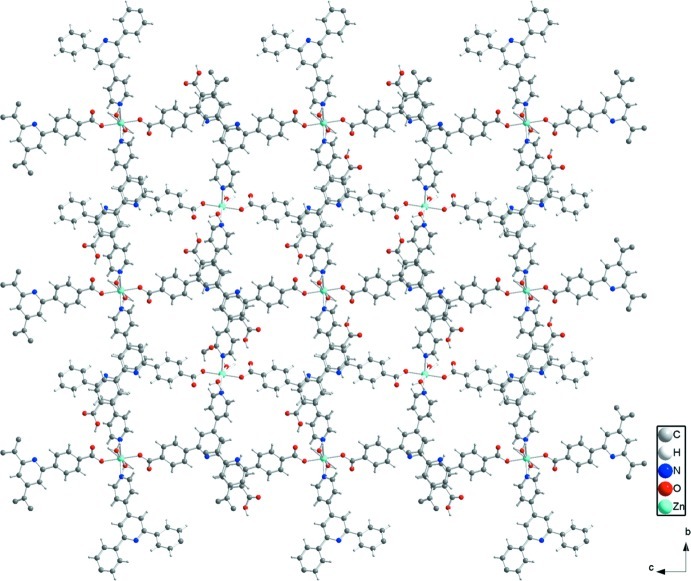
View of the layer structure in the title compound.

## supplementary crystallographic information

### Comment

The coordination chemistry of transition metal complexes is a rapidly growing area due to the ability of the resulting complexes to find applications in materials science with luminescent, magnetic, catalytic, and gas absorption and separation properties (Li *et al.*, 2011; Yang *et al.*, 2007). Multifunctional ligands can link metal ions into one-, two- or three-dimensional structures (Wang *et al.*, 2010). In order to extend the investigations in this field, we used a multifunctional ligand, 4,4'-(4,4'-bipyridine-2,6-diyl)dibenzoic acid (bpydbH_2_), to design and synthesize the title zinc(II) complex and its structure is reported here.

The asymmetric unit of the title complex contains a half Zn^II^ ion, one (bpydbH)^-^ ligand, one aqua ligand and two lattice dimethylformamide (DMF) molecules. As shown in Fig. 1, the Zn^II^ ion, lying on an inversion center, is six-coordinated by two N atoms from two (bpydbH)^-^ ligands, two deprotonated carboxylate O atoms from the other two (bpydbH)^-^ ligands and two aqua ligands, furnishing a slightly distorted octahedral geometry. The bond distances and angles are in the normal range (Song *et al.*, 2012). The Zn nodes are extended by the (bpydbH)^-^ linkers in four directions, forming a layer parallel to (101) (Fig. 2). Intralayer O—H···O hydrogen bonds stabilize the layer. O—H···O hydrogen bonds link the layers and the dimethylformamide solvent molecules. π–π interactions between the pyridine and benzene rings [centroid–centroid distances = 3.7428 (17) and 3.7619 (17) Å] are also present.

### Experimental

Zn(NO_3_)_2_.6H_2_O (0.008 g, 0.025 mmol) and bpydbH_2_ (0.010 g, 0.025 mmol) were suspended in a mixed solution of DMF (4 ml) and H_2_O (0.5 ml). The mixture was heated in a 15 ml Teflon-lined stainless-steel autoclave at 90°C for 3 days. After it was cooled to room temperature slowly, colorless crystals were collected by filtration, washed with DMF and dried in air, with a yield of 59% based on bpydbH_2_.

### Refinement

H atoms on C atoms were positioned geometrically and refined as riding atoms, with C—H = 0.93 (aromatic) and 0.96 (CH_3_) Å and with *U*_iso_(H) = 1.2(1.5 for methyl)*U*_eq_(C). H atoms bonded to O atoms were located in a difference Fourier map and refined with distance restraints of O—H = 0.85 (1) Å and with *U*_iso_(H) = 1.5*U*_eq_(O).

### Crystal data

**Table:**

[Zn(C_24_H_15_N_2_O_4_)_2_(H_2_O)_2_]·4C_3_H_7_NO	*F*(000) = 1240
*M**_r_* = 1184.57	*D*_x_ = 1.388 Mg m^−^^3^
Monoclinic, *P*2_1_/*n*	Mo *K*α radiation, λ = 0.71073 Å
Hall symbol: -P 2yn	Cell parameters from 5607 reflections
*a* = 7.4744 (5) Å	θ = 1.0–26.1°
*b* = 17.7122 (13) Å	µ = 0.51 mm^−^^1^
*c* = 21.4993 (15) Å	*T* = 293 K
β = 95.075 (1)°	Block, colorless
*V* = 2835.1 (3) Å^3^	0.25 × 0.22 × 0.19 mm
*Z* = 2	

### Data collection

**Table:**

Bruker APEXII CCD diffractometer	5606 independent reflections
Radiation source: fine-focus sealed tube	3804 reflections with *I* > 2σ(*I*)
Graphite monochromator	*R*_int_ = 0.044
φ and ω scans	θ_max_ = 26.1°, θ_min_ = 1.9°
Absorption correction: multi-scan (*SADABS*; Bruker, 2001)	*h* = −8→9
*T*_min_ = 0.839, *T*_max_ = 0.915	*k* = −16→21
15534 measured reflections	*l* = −26→21

### Refinement

**Table:**

Refinement on *F*^2^	Primary atom site location: structure-invariant direct methods
Least-squares matrix: full	Secondary atom site location: difference Fourier map
*R*[*F*^2^ > 2σ(*F*^2^)] = 0.053	Hydrogen site location: inferred from neighbouring sites
*wR*(*F*^2^) = 0.158	H atoms treated by a mixture of independent and constrained refinement
*S* = 1.05	*w* = 1/[σ^2^(*F*_o_^2^) + (0.078*P*)^2^ + 1.0747*P*] where *P* = (*F*_o_^2^ + 2*F*_c_^2^)/3
5606 reflections	(Δ/σ)_max_ < 0.001
385 parameters	Δρ_max_ = 0.40 e Å^−^^3^
3 restraints	Δρ_min_ = −0.35 e Å^−^^3^

### Special details

**Table:**

Geometry. All esds (except the esd in the dihedral angle between two l.s. planes) are estimated using the full covariance matrix. The cell esds are taken into account individually in the estimation of esds in distances, angles and torsion angles; correlations between esds in cell parameters are only used when they are defined by crystal symmetry. An approximate (isotropic) treatment of cell esds is used for estimating esds involving l.s. planes.
Refinement. Refinement of F^2^ against ALL reflections. The weighted R-factor wR and goodness of fit S are based on F^2^, conventional R-factors R are based on F, with F set to zero for negative F^2^. The threshold expression of F^2^ > 2sigma(F^2^) is used only for calculating R-factors(gt) etc. and is not relevant to the choice of reflections for refinement. R-factors based on F^2^ are statistically about twice as large as those based on F, and R- factors based on ALL data will be even larger.

### Fractional atomic coordinates and isotropic or equivalent isotropic displacement parameters (Å^2^)

**Table:**

	*x*	*y*	*z*	*U*_iso_*/*U*_eq_	
Zn1	1.0000	0.5000	0.0000	0.03205 (18)	
C1	0.6503 (5)	0.73356 (19)	0.63443 (16)	0.0392 (8)	
C2	0.6897 (4)	0.66640 (18)	0.59720 (14)	0.0331 (7)	
C3	0.6424 (4)	0.59554 (18)	0.61726 (15)	0.0347 (8)	
H3	0.5935	0.5901	0.6553	0.042*	
C4	0.6676 (4)	0.53241 (19)	0.58068 (14)	0.0318 (7)	
H4	0.6355	0.4850	0.5945	0.038*	
C5	0.7403 (4)	0.53927 (17)	0.52353 (13)	0.0266 (6)	
C6	0.7865 (4)	0.61109 (18)	0.50425 (15)	0.0317 (7)	
H6	0.8333	0.6170	0.4659	0.038*	
C7	0.7642 (4)	0.67359 (18)	0.54074 (15)	0.0336 (7)	
H7	0.7993	0.7209	0.5275	0.040*	
C8	0.7645 (4)	0.47318 (18)	0.48218 (14)	0.0275 (7)	
C9	0.7387 (4)	0.39894 (17)	0.50138 (14)	0.0275 (7)	
H9	0.7094	0.3891	0.5417	0.033*	
C10	0.7572 (4)	0.33962 (17)	0.45977 (13)	0.0264 (6)	
C11	0.8034 (4)	0.35839 (17)	0.40001 (13)	0.0288 (7)	
H11	0.8162	0.3207	0.3706	0.035*	
C12	0.8302 (4)	0.43340 (17)	0.38481 (13)	0.0263 (6)	
C13	0.8806 (4)	0.45519 (17)	0.32174 (13)	0.0278 (7)	
C14	0.8268 (4)	0.41227 (18)	0.26902 (14)	0.0329 (7)	
H14	0.7549	0.3700	0.2729	0.039*	
C15	0.8791 (4)	0.43189 (18)	0.21099 (14)	0.0326 (7)	
H15	0.8420	0.4028	0.1762	0.039*	
C16	0.9868 (4)	0.49489 (17)	0.20442 (14)	0.0283 (7)	
C17	1.0387 (4)	0.53839 (18)	0.25678 (14)	0.0321 (7)	
H17	1.1100	0.5809	0.2528	0.038*	
C18	0.9856 (4)	0.51921 (18)	0.31450 (14)	0.0324 (7)	
H18	1.0202	0.5492	0.3490	0.039*	
C19	1.0453 (5)	0.51556 (17)	0.14145 (15)	0.0333 (8)	
C20	0.7223 (4)	0.26062 (17)	0.47603 (13)	0.0266 (6)	
C21	0.7949 (4)	0.19986 (18)	0.44509 (14)	0.0344 (8)	
H21	0.8797	0.2085	0.4168	0.041*	
C22	0.7410 (4)	0.12758 (18)	0.45651 (14)	0.0348 (7)	
H22	0.7923	0.0882	0.4357	0.042*	
C23	0.5550 (4)	0.16840 (18)	0.52842 (14)	0.0326 (7)	
H23	0.4745	0.1578	0.5578	0.039*	
C24	0.6034 (4)	0.24235 (17)	0.52010 (13)	0.0292 (7)	
H24	0.5568	0.2802	0.5439	0.035*	
C25	0.5183 (11)	0.7258 (3)	0.2575 (3)	0.128 (3)	
H25A	0.5527	0.7407	0.2173	0.192*	
H25B	0.5922	0.7515	0.2896	0.192*	
H25C	0.3947	0.7387	0.2606	0.192*	
C26	0.5986 (7)	0.6048 (3)	0.2118 (2)	0.0877 (17)	
H26A	0.6200	0.6397	0.1791	0.132*	
H26B	0.5067	0.5697	0.1970	0.132*	
H26C	0.7073	0.5778	0.2243	0.132*	
C27	0.5016 (7)	0.6134 (3)	0.3160 (2)	0.0726 (13)	
H27	0.5261	0.5620	0.3197	0.087*	
C28	0.8396 (6)	0.1388 (3)	0.6757 (2)	0.0836 (16)	
H28A	0.8060	0.1752	0.7056	0.125*	
H28B	0.8962	0.0964	0.6971	0.125*	
H28C	0.7344	0.1222	0.6506	0.125*	
C29	1.0019 (7)	0.2524 (3)	0.6437 (2)	0.0759 (14)	
H29A	0.9434	0.2711	0.6786	0.114*	
H29B	0.9580	0.2792	0.6066	0.114*	
H29C	1.1291	0.2599	0.6513	0.114*	
C30	1.0301 (5)	0.1338 (2)	0.59015 (18)	0.0506 (10)	
H30	0.9951	0.0835	0.5854	0.061*	
N1	0.8119 (3)	0.49033 (14)	0.42511 (11)	0.0278 (6)	
N2	0.6181 (3)	0.11067 (14)	0.49621 (11)	0.0313 (6)	
N3	0.5410 (4)	0.64597 (18)	0.26498 (14)	0.0501 (8)	
N4	0.9645 (4)	0.1733 (2)	0.63570 (15)	0.0520 (8)	
O1	0.9686 (3)	0.48245 (12)	0.09483 (9)	0.0361 (6)	
O2	1.1689 (3)	0.56380 (13)	0.13980 (10)	0.0432 (6)	
O3	0.7257 (4)	0.79565 (15)	0.61423 (13)	0.0569 (7)	
H3A	0.683 (6)	0.8386 (13)	0.624 (2)	0.085*	
O4	0.5576 (4)	0.73346 (15)	0.67822 (12)	0.0544 (7)	
O5	0.4357 (5)	0.64314 (19)	0.35968 (14)	0.0839 (11)	
O6	1.1334 (4)	0.15890 (17)	0.55402 (13)	0.0590 (8)	
O1W	1.2630 (3)	0.54461 (14)	0.02156 (11)	0.0416 (6)	
H1A	1.299 (5)	0.5844 (14)	0.0047 (18)	0.062*	
H1B	1.278 (5)	0.559 (2)	0.0592 (8)	0.062*	

### Atomic displacement parameters (Å^2^)

**Table:**

	*U*^11^	*U*^22^	*U*^33^	*U*^12^	*U*^13^	*U*^23^
Zn1	0.0519 (3)	0.0240 (3)	0.0219 (3)	0.0006 (2)	0.0128 (2)	0.0000 (2)
C1	0.050 (2)	0.033 (2)	0.0353 (19)	0.0030 (16)	0.0075 (16)	−0.0027 (15)
C2	0.0362 (17)	0.0336 (18)	0.0293 (17)	0.0046 (14)	0.0013 (14)	−0.0048 (14)
C3	0.0421 (18)	0.0354 (19)	0.0278 (17)	0.0034 (15)	0.0100 (14)	0.0005 (14)
C4	0.0413 (18)	0.0293 (17)	0.0254 (16)	0.0013 (14)	0.0066 (14)	0.0021 (13)
C5	0.0270 (15)	0.0278 (17)	0.0248 (16)	0.0007 (13)	0.0013 (12)	0.0009 (12)
C6	0.0361 (17)	0.0308 (18)	0.0291 (17)	−0.0009 (14)	0.0081 (14)	−0.0001 (13)
C7	0.0378 (18)	0.0275 (17)	0.0360 (18)	−0.0006 (14)	0.0060 (15)	0.0006 (14)
C8	0.0272 (15)	0.0292 (16)	0.0266 (16)	−0.0020 (13)	0.0054 (13)	−0.0014 (13)
C9	0.0295 (16)	0.0309 (17)	0.0228 (15)	−0.0004 (13)	0.0057 (13)	0.0016 (12)
C10	0.0298 (15)	0.0284 (16)	0.0214 (15)	0.0015 (13)	0.0046 (12)	0.0016 (12)
C11	0.0371 (17)	0.0282 (17)	0.0217 (15)	0.0012 (13)	0.0063 (13)	−0.0005 (12)
C12	0.0317 (16)	0.0287 (17)	0.0187 (14)	−0.0002 (13)	0.0039 (12)	0.0017 (12)
C13	0.0338 (16)	0.0274 (17)	0.0229 (15)	0.0020 (13)	0.0074 (13)	0.0034 (12)
C14	0.0452 (19)	0.0290 (17)	0.0261 (16)	−0.0059 (14)	0.0118 (14)	0.0008 (13)
C15	0.0480 (19)	0.0296 (18)	0.0211 (15)	0.0010 (14)	0.0075 (14)	0.0008 (13)
C16	0.0390 (17)	0.0244 (16)	0.0226 (15)	0.0038 (13)	0.0102 (13)	0.0026 (12)
C17	0.0437 (18)	0.0272 (17)	0.0263 (16)	−0.0049 (14)	0.0081 (14)	0.0032 (13)
C18	0.0448 (18)	0.0322 (18)	0.0209 (16)	−0.0038 (14)	0.0065 (14)	−0.0012 (12)
C19	0.0482 (19)	0.0265 (18)	0.0268 (17)	0.0052 (15)	0.0115 (15)	0.0043 (13)
C20	0.0333 (16)	0.0241 (16)	0.0229 (15)	0.0006 (13)	0.0054 (12)	0.0001 (12)
C21	0.0439 (19)	0.0327 (18)	0.0286 (17)	−0.0002 (15)	0.0144 (15)	0.0001 (13)
C22	0.0481 (19)	0.0285 (17)	0.0296 (17)	0.0029 (15)	0.0132 (15)	−0.0025 (13)
C23	0.0461 (19)	0.0294 (17)	0.0240 (16)	0.0000 (14)	0.0124 (14)	0.0014 (13)
C24	0.0406 (17)	0.0264 (16)	0.0213 (15)	−0.0009 (13)	0.0063 (13)	−0.0003 (12)
C25	0.226 (8)	0.052 (4)	0.107 (5)	0.005 (4)	0.016 (5)	0.023 (3)
C26	0.084 (3)	0.127 (5)	0.055 (3)	0.038 (3)	0.025 (3)	0.002 (3)
C27	0.103 (4)	0.055 (3)	0.064 (3)	0.011 (3)	0.032 (3)	0.009 (2)
C28	0.078 (3)	0.116 (5)	0.061 (3)	−0.027 (3)	0.031 (3)	−0.016 (3)
C29	0.092 (3)	0.065 (3)	0.073 (3)	0.005 (3)	0.020 (3)	−0.018 (2)
C30	0.054 (2)	0.051 (2)	0.047 (2)	0.0043 (19)	0.0038 (19)	−0.0033 (18)
N1	0.0320 (13)	0.0289 (15)	0.0235 (13)	−0.0014 (11)	0.0069 (11)	0.0025 (10)
N2	0.0468 (16)	0.0251 (14)	0.0232 (13)	−0.0013 (12)	0.0103 (12)	0.0005 (10)
N3	0.059 (2)	0.051 (2)	0.0425 (18)	0.0119 (16)	0.0188 (15)	0.0105 (15)
N4	0.0484 (18)	0.063 (2)	0.0460 (19)	0.0022 (16)	0.0137 (15)	−0.0078 (16)
O1	0.0617 (15)	0.0290 (13)	0.0190 (11)	−0.0026 (10)	0.0118 (11)	−0.0013 (9)
O2	0.0644 (16)	0.0364 (14)	0.0311 (12)	−0.0112 (12)	0.0170 (11)	0.0010 (10)
O3	0.0751 (19)	0.0377 (16)	0.0612 (17)	0.0008 (14)	0.0256 (15)	−0.0095 (13)
O4	0.0735 (18)	0.0479 (17)	0.0439 (15)	0.0008 (14)	0.0177 (14)	−0.0080 (12)
O5	0.134 (3)	0.071 (2)	0.0527 (18)	0.008 (2)	0.044 (2)	−0.0085 (16)
O6	0.0602 (17)	0.065 (2)	0.0548 (17)	0.0113 (14)	0.0242 (14)	−0.0028 (14)
O1W	0.0544 (15)	0.0381 (15)	0.0345 (13)	−0.0061 (12)	0.0165 (12)	−0.0018 (11)

### Geometric parameters (Å, º)

**Table:**

Zn1—O1	2.096 (2)	C18—H18	0.9300
Zn1—O1^i^	2.096 (2)	C19—O1	1.255 (4)
Zn1—O1W	2.131 (3)	C19—O2	1.261 (4)
Zn1—O1W^i^	2.131 (3)	C20—C24	1.393 (4)
Zn1—N2^ii^	2.154 (3)	C20—C21	1.400 (4)
Zn1—N2^iii^	2.154 (3)	C21—C22	1.371 (4)
C1—O4	1.217 (4)	C21—H21	0.9300
C1—O3	1.326 (4)	C22—N2	1.342 (4)
C1—C2	1.478 (4)	C22—H22	0.9300
C2—C3	1.383 (5)	C23—N2	1.344 (4)
C2—C7	1.385 (4)	C23—C24	1.375 (4)
C3—C4	1.389 (4)	C23—H23	0.9300
C3—H3	0.9300	C24—H24	0.9300
C4—C5	1.392 (4)	C25—N3	1.431 (6)
C4—H4	0.9300	C25—H25A	0.9600
C5—C6	1.391 (4)	C25—H25B	0.9600
C5—C8	1.490 (4)	C25—H25C	0.9600
C6—C7	1.375 (4)	C26—N3	1.453 (5)
C6—H6	0.9300	C26—H26A	0.9600
C7—H7	0.9300	C26—H26B	0.9600
C8—N1	1.342 (4)	C26—H26C	0.9600
C8—C9	1.397 (4)	C27—O5	1.217 (5)
C9—C10	1.395 (4)	C27—N3	1.297 (5)
C9—H9	0.9300	C27—H27	0.9300
C10—C11	1.400 (4)	C28—N4	1.458 (5)
C10—C20	1.471 (4)	C28—H28A	0.9600
C11—C12	1.387 (4)	C28—H28B	0.9600
C11—H11	0.9300	C28—H28C	0.9600
C12—N1	1.344 (4)	C29—N4	1.436 (5)
C12—C13	1.490 (4)	C29—H29A	0.9600
C13—C14	1.394 (4)	C29—H29B	0.9600
C13—C18	1.396 (4)	C29—H29C	0.9600
C14—C15	1.384 (4)	C30—O6	1.226 (4)
C14—H14	0.9300	C30—N4	1.333 (5)
C15—C16	1.390 (4)	C30—H30	0.9300
C15—H15	0.9300	N2—Zn1^iv^	2.154 (3)
C16—C17	1.391 (4)	O3—H3A	0.86 (1)
C16—C19	1.504 (4)	O1W—H1A	0.85 (1)
C17—C18	1.378 (4)	O1W—H1B	0.85 (1)
C17—H17	0.9300		
			
O1—Zn1—O1^i^	180.0	C17—C18—C13	120.7 (3)
O1—Zn1—O1W	91.38 (9)	C17—C18—H18	119.7
O1^i^—Zn1—O1W	88.62 (9)	C13—C18—H18	119.7
O1—Zn1—O1W^i^	88.62 (9)	O1—C19—O2	125.3 (3)
O1^i^—Zn1—O1W^i^	91.38 (9)	O1—C19—C16	117.1 (3)
O1W—Zn1—O1W^i^	180.0	O2—C19—C16	117.5 (3)
O1—Zn1—N2^ii^	89.05 (9)	C24—C20—C21	116.2 (3)
O1^i^—Zn1—N2^ii^	90.95 (9)	C24—C20—C10	121.3 (3)
O1W—Zn1—N2^ii^	88.43 (10)	C21—C20—C10	122.3 (3)
O1W^i^—Zn1—N2^ii^	91.57 (10)	C22—C21—C20	120.1 (3)
O1—Zn1—N2^iii^	90.95 (9)	C22—C21—H21	120.0
O1^i^—Zn1—N2^iii^	89.05 (9)	C20—C21—H21	120.0
O1W—Zn1—N2^iii^	91.57 (10)	N2—C22—C21	123.4 (3)
O1W^i^—Zn1—N2^iii^	88.43 (10)	N2—C22—H22	118.3
N2^ii^—Zn1—N2^iii^	180.0	C21—C22—H22	118.3
O4—C1—O3	122.8 (3)	N2—C23—C24	123.3 (3)
O4—C1—C2	124.9 (3)	N2—C23—H23	118.3
O3—C1—C2	112.3 (3)	C24—C23—H23	118.3
C3—C2—C7	119.4 (3)	C23—C24—C20	120.1 (3)
C3—C2—C1	119.5 (3)	C23—C24—H24	120.0
C7—C2—C1	121.0 (3)	C20—C24—H24	120.0
C2—C3—C4	120.2 (3)	N3—C25—H25A	109.5
C2—C3—H3	119.9	N3—C25—H25B	109.5
C4—C3—H3	119.9	H25A—C25—H25B	109.5
C3—C4—C5	120.8 (3)	N3—C25—H25C	109.5
C3—C4—H4	119.6	H25A—C25—H25C	109.5
C5—C4—H4	119.6	H25B—C25—H25C	109.5
C6—C5—C4	118.0 (3)	N3—C26—H26A	109.5
C6—C5—C8	119.6 (3)	N3—C26—H26B	109.5
C4—C5—C8	122.4 (3)	H26A—C26—H26B	109.5
C7—C6—C5	121.4 (3)	N3—C26—H26C	109.5
C7—C6—H6	119.3	H26A—C26—H26C	109.5
C5—C6—H6	119.3	H26B—C26—H26C	109.5
C6—C7—C2	120.3 (3)	O5—C27—N3	126.7 (5)
C6—C7—H7	119.9	O5—C27—H27	116.6
C2—C7—H7	119.9	N3—C27—H27	116.6
N1—C8—C9	122.5 (3)	N4—C28—H28A	109.5
N1—C8—C5	115.0 (3)	N4—C28—H28B	109.5
C9—C8—C5	122.4 (3)	H28A—C28—H28B	109.5
C10—C9—C8	119.7 (3)	N4—C28—H28C	109.5
C10—C9—H9	120.1	H28A—C28—H28C	109.5
C8—C9—H9	120.1	H28B—C28—H28C	109.5
C9—C10—C11	117.1 (3)	N4—C29—H29A	109.5
C9—C10—C20	122.3 (3)	N4—C29—H29B	109.5
C11—C10—C20	120.5 (3)	H29A—C29—H29B	109.5
C12—C11—C10	119.8 (3)	N4—C29—H29C	109.5
C12—C11—H11	120.1	H29A—C29—H29C	109.5
C10—C11—H11	120.1	H29B—C29—H29C	109.5
N1—C12—C11	122.8 (3)	O6—C30—N4	124.6 (4)
N1—C12—C13	116.1 (3)	O6—C30—H30	117.7
C11—C12—C13	121.1 (3)	N4—C30—H30	117.7
C14—C13—C18	118.5 (3)	C8—N1—C12	118.0 (3)
C14—C13—C12	121.4 (3)	C22—N2—C23	116.7 (3)
C18—C13—C12	120.1 (3)	C22—N2—Zn1^iv^	122.2 (2)
C15—C14—C13	120.8 (3)	C23—N2—Zn1^iv^	120.6 (2)
C15—C14—H14	119.6	C27—N3—C25	120.0 (4)
C13—C14—H14	119.6	C27—N3—C26	123.2 (4)
C14—C15—C16	120.3 (3)	C25—N3—C26	116.6 (4)
C14—C15—H15	119.8	C30—N4—C29	121.4 (4)
C16—C15—H15	119.8	C30—N4—C28	120.6 (4)
C15—C16—C17	119.0 (3)	C29—N4—C28	117.8 (4)
C15—C16—C19	120.3 (3)	C19—O1—Zn1	128.7 (2)
C17—C16—C19	120.7 (3)	C1—O3—H3A	119 (3)
C18—C17—C16	120.7 (3)	Zn1—O1W—H1A	122 (3)
C18—C17—H17	119.7	Zn1—O1W—H1B	111 (3)
C16—C17—H17	119.7	H1A—O1W—H1B	98 (4)
			
O4—C1—C2—C3	−9.9 (5)	C19—C16—C17—C18	−179.7 (3)
O3—C1—C2—C3	171.1 (3)	C16—C17—C18—C13	0.8 (5)
O4—C1—C2—C7	166.5 (3)	C14—C13—C18—C17	−1.7 (5)
O3—C1—C2—C7	−12.6 (5)	C12—C13—C18—C17	177.6 (3)
C7—C2—C3—C4	−0.7 (5)	C15—C16—C19—O1	12.2 (4)
C1—C2—C3—C4	175.7 (3)	C17—C16—C19—O1	−167.6 (3)
C2—C3—C4—C5	−0.1 (5)	C15—C16—C19—O2	−166.8 (3)
C3—C4—C5—C6	−0.1 (5)	C17—C16—C19—O2	13.4 (5)
C3—C4—C5—C8	−178.2 (3)	C9—C10—C20—C24	26.9 (4)
C4—C5—C6—C7	1.1 (4)	C11—C10—C20—C24	−150.4 (3)
C8—C5—C6—C7	179.3 (3)	C9—C10—C20—C21	−158.1 (3)
C5—C6—C7—C2	−1.9 (5)	C11—C10—C20—C21	24.6 (4)
C3—C2—C7—C6	1.7 (5)	C24—C20—C21—C22	3.3 (4)
C1—C2—C7—C6	−174.6 (3)	C10—C20—C21—C22	−171.9 (3)
C6—C5—C8—N1	−7.2 (4)	C20—C21—C22—N2	0.7 (5)
C4—C5—C8—N1	170.9 (3)	N2—C23—C24—C20	0.6 (5)
C6—C5—C8—C9	173.1 (3)	C21—C20—C24—C23	−3.9 (4)
C4—C5—C8—C9	−8.8 (4)	C10—C20—C24—C23	171.3 (3)
N1—C8—C9—C10	−1.9 (4)	C9—C8—N1—C12	1.8 (4)
C5—C8—C9—C10	177.7 (3)	C5—C8—N1—C12	−177.9 (2)
C8—C9—C10—C11	0.7 (4)	C11—C12—N1—C8	−0.4 (4)
C8—C9—C10—C20	−176.6 (3)	C13—C12—N1—C8	179.2 (2)
C9—C10—C11—C12	0.5 (4)	C21—C22—N2—C23	−4.0 (5)
C20—C10—C11—C12	177.9 (3)	C21—C22—N2—Zn1^iv^	168.0 (2)
C10—C11—C12—N1	−0.7 (5)	C24—C23—N2—C22	3.4 (5)
C10—C11—C12—C13	179.6 (3)	C24—C23—N2—Zn1^iv^	−168.8 (2)
N1—C12—C13—C14	−150.2 (3)	O5—C27—N3—C25	4.1 (8)
C11—C12—C13—C14	29.5 (4)	O5—C27—N3—C26	−172.1 (5)
N1—C12—C13—C18	30.6 (4)	O6—C30—N4—C29	4.2 (6)
C11—C12—C13—C18	−149.7 (3)	O6—C30—N4—C28	178.8 (4)
C18—C13—C14—C15	1.2 (5)	O2—C19—O1—Zn1	−5.2 (5)
C12—C13—C14—C15	−178.1 (3)	C16—C19—O1—Zn1	175.85 (19)
C13—C14—C15—C16	0.1 (5)	O1W—Zn1—O1—C19	24.7 (3)
C14—C15—C16—C17	−1.0 (5)	O1W^i^—Zn1—O1—C19	−155.3 (3)
C14—C15—C16—C19	179.3 (3)	N2^ii^—Zn1—O1—C19	113.1 (3)
C15—C16—C17—C18	0.5 (5)	N2^iii^—Zn1—O1—C19	−66.9 (3)

Symmetry codes: (i) −*x*+2, −*y*+1, −*z*; (ii) *x*+1/2, −*y*+1/2, *z*−1/2; (iii) −*x*+3/2, *y*+1/2, −*z*+1/2; (iv) −*x*+3/2, *y*−1/2, −*z*+1/2.

### Hydrogen-bond geometry (Å, º)

**Table:**

*D*—H···*A*	*D*—H	H···*A*	*D*···*A*	*D*—H···*A*
O1*W*—H1*A*···O6^v^	0.85 (1)	1.93 (2)	2.750 (4)	164 (4)
O1*W*—H1*B*···O2	0.85 (1)	1.98 (2)	2.718 (3)	145 (4)
O3—H3*A*···O2^vi^	0.86 (1)	1.77 (2)	2.592 (3)	161 (5)

Symmetry codes: (v) −*x*+5/2, *y*+1/2, −*z*+1/2; (vi) *x*−1/2, −*y*+3/2, *z*+1/2.
